# An emerging role for microRNAs in NF1 tumorigenesis

**DOI:** 10.1186/1479-7364-6-23

**Published:** 2012-11-17

**Authors:** Ashni Sedani, David N Cooper, Meena Upadhyaya

**Affiliations:** 1 Institute of Medical Genetics, School of Medicine, Cardiff University, Heath Park, Cardiff, CF14 4XN, UK

**Keywords:** MicroRNAs, Neurofibromatosis type 1, Malignant peripheral nerve sheath tumours, Tumorigenesis

## Abstract

MicroRNAs (miRNAs) are a class of non-coding RNA, which have recently been shown to have a wide variety of regulatory functions in relation to gene expression. Since their identification nearly 20 years ago, miRNAs have been found to play an important role in cancer, including in neurofibromatosis type 1 (NF1)-associated tumours. NF1 is the most commonly inherited tumour predisposition syndrome and can lead to malignancy via the development of malignant peripheral nerve sheath tumours (MPNSTs). Although the mechanisms by which benign neurofibromas develop into MPNSTs still remain to be elucidated, it is becoming increasingly clear that miRNAs play a key role in this process and have the potential to be used as both diagnostic and prognostic markers of tumorigenesis.

## Introduction

miRNAs constitute a category of small RNAs, ranging between 19 and 25 nucleotides in length, which are generated from endogenous hairpin-shaped transcripts. They function predominantly as post-transcriptional regulators of gene expression by hybridizing to the 3^′^ untranslated regions (3^′^UTRs) of target mRNA molecules [1,2], leading to translational repression or cleavage of the target mRNA [3]. The first described miRNA, known as *lin4*, was discovered in *Caenorhabditis elegans* by Lee et al. in 1993 [4]. This was later followed by the discovery of *let7*[5], which was found to be highly conserved across many different species [6], indicating the widespread existence of miRNAs in eukaryotes. More than 1,500 human miRNA sequences have been reported in the human genome to date (miRBase; http://www.mirbase.org/), of which 50% have been found to occur within clusters, targeting either the same or different genes within the same biological pathway [7]. It has become apparent that miRNAs regulate the expression of at least 30% of all protein-coding genes in mammalian genomes [8], underlining their likely role in human genetic disease. Novel techniques developed for the identification of miRNAs have been instrumental in the rapid progress made by miRNA research. miRNAs play an important role in the development and progression of cancer. With an ever-increasing number of studies focusing on the roles of miRNAs in cancer, whether operating as oncogenes or tumour suppressor genes, our understanding of the way in which miRNAs function is steadily improving, and hence the number of potential therapeutic applications for miRNAs should also increase.

In this review, the structure, function and biogenesis of miRNA molecules are discussed in reference to the recent developments that focus upon the role of miRNAs in relation to neurofibromatosis type 1.

### miRNA biogenesis, export and function

**Figure 1:**
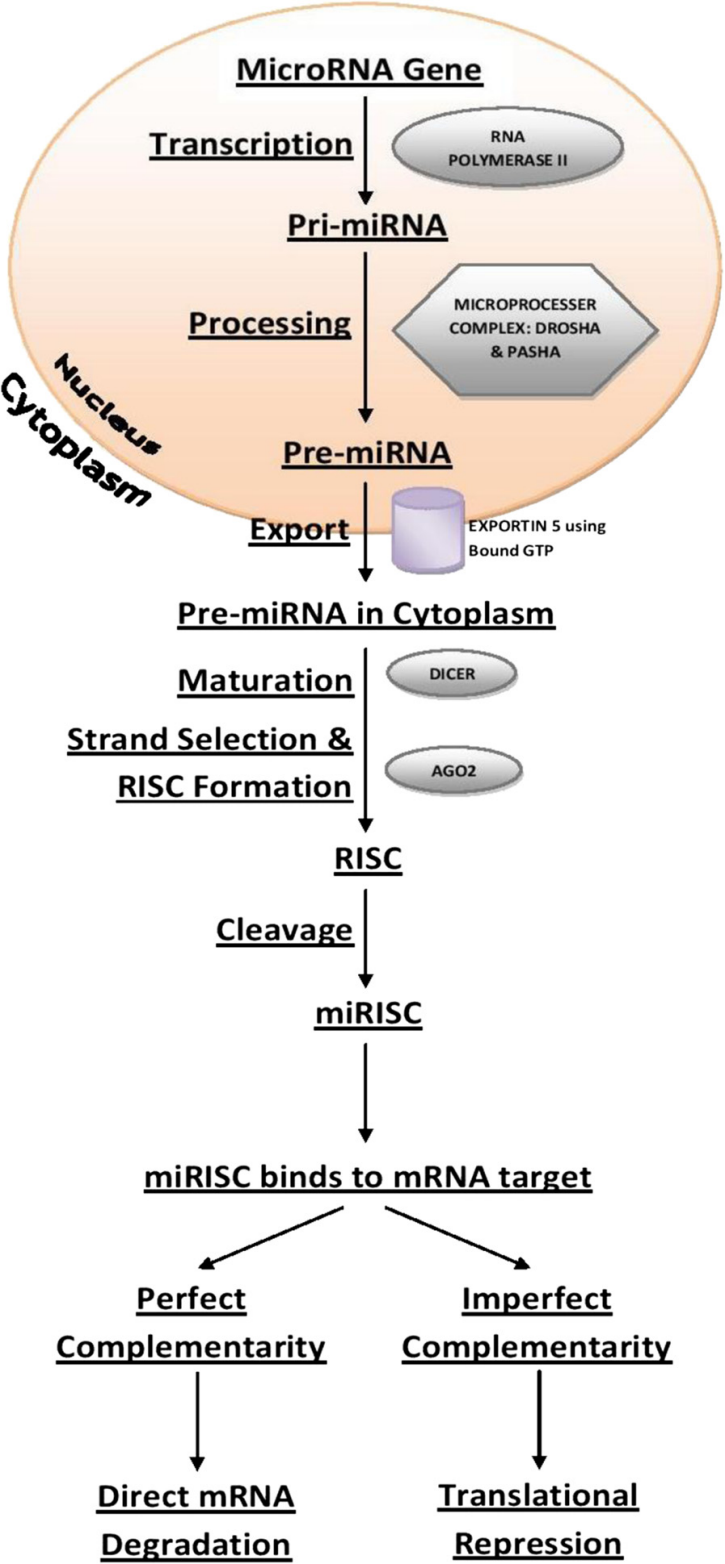
**The flowchart highlights the processes involved in the following miRNA biogenesis: (1) The process begins inside the nucleus, where RNA polymerase II or III initiates the transcription of miRNA-coding genes to produce ‘pri-microRNA’s**[10,11]. (2) A microprocessor complex comprising both Drosha, an RNase III class enzyme, and Pasha, identifies and cleaves pre-microRNAs generating pre-microRNAs [12-14]. (3) Pre-microRNA molecules are transferred to the cytoplasm through exportin-5-mediated transport, which uses GTP that is bound to the Ran protein. The function of exportin-5 is dependent upon the GTP-bound form of the Ran co-factor for specific binding to the corresponding substrates. Therefore, this process comprises the hydrolysis of Ran-GTP to Ran-GDP, via the Ran GTPase-activating protein in the cytoplasm [15,16]. (4) In the cytoplasm, Dicer acts to cleave pre-miRNA molecules and, with the action of Argonaute 2 which is required for miRNA-induced silencing, forms an RNA-induced silencing complex (RISC) leading to the creation of a miRNA-induced silencing complex (miRISC). (5) Interaction between the miRNA and its target mRNA in the miRISC can cause either translational repression or miRNA degradation depending upon the degree of complementarity.

The process of miRNA biogenesis involves miRNA transcription, the transport of the miRNAs to the cytoplasm and subsequent maturation. miRNA biogenesis has been reviewed in detail by Ladomery et al. [9] and will not be specifically discussed here. However, the basic process has been summarised in Figure 1.

The mechanism by which mature miRNAs regulate gene expression depends upon the level of complementarity that exists between the miRNA and its target mRNA [17]. Gene silencing can be effected either by mRNA degradation or by inhibiting the mRNA from being translated. It has been shown that if there is complete complementarity between the miRNA and target mRNA sequence, then Argonaute 2, a member of the Argonaute protein family, can carry out strand selection and separation causing an RNA-induced silencing complex (RISC) to bind to its mRNA target, leading to mRNA degradation which in turn impacts upon translation [18]. If, however, the complementarity is imperfect, as seen in most cases, translational repression ensues [19]. Therefore, the main function of miRNAs appears to be in the context of gene regulation via mRNA degradation. Rarely, miRNAs can also bring about histone modification and DNA methylation of gene promoter regions, thereby indirectly altering the expression of target genes [20,21].

## Methodologies

miRNA expression profiles can be used both to categorize different types of cancer and to identify miRNA markers that can help in making prognostic predictions [22]. Initially, northern blotting was used to detect pre-miRNAs and mature miRNAs and to provide information about the regulation of the enzymes involved in miRNA biogenesis [23]. Real-time polymerase chain reaction (PCR) and microarray techniques have also been tailored to detect the expression of pre-miRNAs and mature miRNAs using either SYBR Green dye or TaqMan probes (Sigma-Aldrich Corporation, St. Louis, MO, USA) [24]. Further, Liu et al. in 2004 [25] used oligonucleotide microchips to identify distinct miRNA expression patterns in breast cancer and B cell chronic lymphocytic leukaemia. Nevertheless, miRNA microarray techniques are constantly being improved; thus, Neely et al. in 2006 [26] developed modified oligonucleotide arrays where probes for each miRNA were designed and labelled with different fluorophores whereas Lu et al. in 2005 [27] used a flow cytometry-based method analysis to demonstrate expression of miRNAs from a range of different samples. Both methods allowed the quantification of miRNA expression, thereby making cancer diagnosis through miRNA analysis more specific and accurate. The advantage of using miRNAs as markers instead of mRNAs lies in their ability to aid the classification of poorly differentiated cancers, as evidenced by the study by Rosenfeld et al. in 2008 [28], suggesting that miRNAs can be used to correctly identify the tumour tissue of origin.

The potential role of miRNAs in pathology has been studied by looking not only at their single nucleotide polymorphisms (SNPs) but also at their copy number variation (CNV). Functional polymorphisms in the 3^′^UTRs of miRNA genes have been reported to be associated with cancer by virtue of their altering gene expression. SNPs may occur in miRNA biogenesis pathway genes, primary miRNAs, pre-miRNAs or mature miRNA sequences and have the potential to affect the efficiency of miRNA binding to their target sites or alternatively can create or disrupt miRNA binding sites [29]. In a recent study, Gong et al. [30] identified 48 SNPs in human miRNA seed regions and a large number of SNPs in 3^′^UTRs with the potential to either disturb or create miRNA target interactions. They confirmed the loss-of-function and gain-of-function SNPs by luciferase assay.

Zhang et al. in 2006 [31] found that a large proportion of loci containing miRNA genes exhibited somatic copy number alterations and, amongst the three cancer types, they identified 41 miRNAs with copy number changes. These miRNA copy number changes also correlated with the levels of miRNA expression in these cancers. High frequency copy number changes were also noted in the genes encoding Dicer (*DICER1*) and Argonaute 2 (*EIF2C2*), both of which are required for miRNA biogenesis. These findings are compatible with the view that somatic miRNA copy number changes are common in cancer and could potentially account for at least a proportion of the miRNA deregulation found in many tumour types. Using bioinformatic tools, Marcinkowska et al. in 2011 [32] have demonstrated that miRNA loci are under-represented in highly polymorphic and well-validated CNV regions. Thus, CNV-miRNAs represent functional variants of potential importance for genotype/phenotype association studies.

### miRNAs in neurofibromatosis type 1

Neurofibromatosis type 1 (NF1), a familial tumour predisposition syndrome, is characterised by the growth of benign and malignant tumours involving the peripheral and central nervous system. NF1 results from inactivating germline mutations of the *NF1* gene located at 17q11.2 [33]. Most NF1 patients develop multiple benign cutaneous neurofibromas, with approximately 30% to 50% of patients also developing larger plexiform neurofibromas. About 10% of patients eventually develop malignant peripheral nerve sheath tumours (MPNSTs), which are aggressive tumours that pose significant diagnostic and therapeutic challenges. Half of all MPNSTs diagnosed occur in association with NF1, with affected patients exhibiting a poor prognosis. With no effective treatment available, radical surgery and chemo and radiotherapy are required to reduce tumour recurrence and metastasis and prolong patient survival.

The process of MPNST pathogenesis is poorly understood owing to its complex histopathology and the underlying molecular mechanisms. Biallelic *NF1* gene inactivation is essential for tumour development. However, it is now known that additional molecular changes and the tumour micro-environment are associated with the progression of this type of tumour. There is currently no defined molecular signature for MPNST development. Constitutive activation of several critical cell signalling cascades also occurs in MPNSTs. Multidisciplinary collaborative efforts are clearly essential to fully decipher both the complex molecular basis of MPNST development and to define potential therapeutic targets.

A general characteristic of cancer cells is uncontrolled growth and, in many cases, miRNAs can either promote or suppress this growth. Previous investigations have shown that miRNAs have key functions in the development of cancer, in relation to a range of different cellular processes including cell differentiation, developmental control, neural development, cell proliferation, apoptosis [34,35] and organ development [3]. Thus, the role of a given miRNA in cancer pathogenesis can be categorised as being either of oncogene or tumour suppressor gene nature. Moreover, it is thought that >50% of miRNA-coding genes are located in cancer-associated genomic regions or in fragile sites [36]. It is now known that miRNAs are associated with a range of different cancers, including chronic leukaemias, acute leukaemias and myelodysplastic syndromes, lymphomas, multiple myeloma, hepatocellular carcinoma, breast, lung and colon cancer, amongst others.

Several research groups have studied the role of miRNA in NF1-associated malignancy. Here we focus on miRNAs miR-29c, miR-34a, miR-214, miR-10b, miR204 and miR-21 which have previously been directly implicated in NF1 tumorigenesis (summarized in Table 1).

**Table 1:** **Functionally characterised miRNAs identified in NF1**-**MPNSTs**

**miRNA**	**Change in expression in MPNSTs**	**Tumour suppressor or oncogene role**	**Reference**
miR-34a	Down-regulated	Tumour suppressor	[37]
miR-214	Over-expressed	Oncogene	[37]
miR-10b	Over-expressed	Oncogene	[37,38]
miR-29c	Down-regulated	Tumour suppressor	[39]
miR204	Down-regulated	Tumour suppressor	[40]
miRNA-21	Over-expressed	Oncogene	[41]

### miR-34a

One of the earliest studies of miRNAs in NF1 was carried out by Subramanian et al. [37]. Their genome-wide transcriptome analyses revealed that the development of malignancy from neurofibroma to MPNST correlates with the loss of expression of a number of genes rather than an increase in expression. This subsequently led to miRNA expression profiling which demonstrated the down-regulation of miR-34a in most MPNSTs as compared to neurofibromas. This loss of miR-34a could contribute to the development of malignancy. miR-34 is known to be a direct target of p53 in a variety of neoplasms including colorectal carcinoma and neuroblastoma in which lower levels of this miRNA are associated with the suppression of apoptosis. Forced expression of miR-34a in MPNST cell lines that are deficient in miR-34a leads to increased apoptosis whereas the forced expression of p53 in the same cell line results in a similar effect, indicating that the expression of p53 results in apoptosis through a mechanism involving miR-34a. The study of Subramanian et al. in 2010 also revealed an increase in the expression levels of a further nine miRNAs, following induced expression of p53 in MPNSTs. Interestingly, six of these (including miR-638, miR-373, miR-492, miR-126, miR-140 and miR-491), have been shown to be involved in promoting tumorigenic processes such as invasion, proliferation and metastasis.

Using a systems biology approach, Lee et al. [42] identified a regulatory network which involved the E2F family: E2F7/E2F8 in several cell cycle-related gene modules. miR-34a over-expression was found to lead to increased activity of E2F7/E2F8 transcription factors and cell cycle genes. These transcription factors are disproportionately associated with DNA damage repair, cell growth and development [42,43], and the altered expression levels of this family of transcription factors has been noted in cancerous cells [44], indicating that miR-34a could be a target for therapeutic intervention. As E2F transcription factors are involved in transcriptional regulation as well as in DNA repair and cell proliferation, it is possible that an alteration in the expression of activators and suppressors of E2F7 and E2F8 transcription may play a role in the development of MPNSTs. However, recent data by Presneau et al. [39] failed to confirm the work by Subramanian et al. [37] regarding miR-34a. This was deemed to be due either to differences in the methodologies used or to the use of different groups of patients based on differences in NF1 diagnostic criteria. Recent reports indicate that miR-34a has also been shown to act as a tumour suppressor through its dysregulation in a number of other carcinomas including glioblastoma multiforme tumours [45], breast cancer [46], head and neck squamous cell carcinoma [47] and osteosarcoma [48], suggesting that it could represent a significant gene target in cancer therapeutics.

### miR-214

Subramanian et al. [37] identified miR-214 as having the highest expression level of all miRNAs screened in their study of NF1-MPNSTs. miR-214 had previously been shown to be expressed at an increased level in the blood of breast and ovarian cancer patients, indicating that it could be used as an indicator of malignancy [49,50]. Yang et al. [50] proposed that miR-214 induces cell survival and cisplatin resistance through targeting the 3^′^UTR of the *PTEN* gene, which leads to down-regulation of the PTEN protein and the activation of the Akt pathway, thereby promoting cell survival. More recently, Peng et al. [51] found that miR-214 can inhibit cancer cell proliferation and migration by targeting *GALNT7.* This is supported by data from Shih et al. [52], who showed that miR-214 down-regulation can contribute to tumour angiogenesis and is associated with increased tumour recurrence and a poor prognosis. In their study, Subramanian et al. [37] also identified a metastasis-promoting factor, *TWIST1*, as promoting expression of miR-214. *TWIST1* had previously been noted to increase metastasis in a number of different carcinomas, but its exact role in NF1 tumorigenesis has not yet been elucidated. Taken together with the above data, it is suggested that *TWIST1* may regulate miR-214 expression thereby contributing to tumorigenesis [53].

### miR-10b

By inhibiting the expression of miR-10b in NF1-MPNSTs, Subramanian et al. [37] demonstrated decreased cell proliferation and migration, indicating an important function for miR-10b in the transformation of benign NF1-associated neurofibromas to MPNSTs. These workers also showed that the expression of miR-10b is considerably higher in NF1-MPNST cell lines, NF1-MPNST tumour tissues and primary Schwann cells by comparison with benign neurofibromas. This suggests that miR-10b could act as a biomarker to differentiate between NF1-MPNSTs and neurofibromas. miR-10b belongs to the miR-10 cluster of miRNAs and is highly conserved between different species. Its location within the Hox gene clusters suggests that it could share the same mechanisms of regulation as the Hox genes [54]. Supporting this notion that over-expressed miRNAs of the miR-10 family may play a role in cancer, miR-10b has also been found to be overexpressed in B cell chronic lymphocytic leukaemia [36] as well as in acute myeloid leukaemias associated with mutations in the *NPM1* gene [55].

miR-10b expression has also been shown as enhanced by increased expression of *TWIST1* in MPNST cells [56]. Chai et al. [38] have suggested that it is the increased expression of *TWIST1* that may cause increased expression of miR-10b in the MPNSTs of NF1 patients. In their study, which compared MPNST cell lines from NF1 patients with those from sporadic tumours, Chai et al. [38] identified miR-10b, miR-155 and miR-335 as being over-expressed and Let-7a and Let-7b as being under-expressed in the NF1-MPNST cell lines. Further investigation showed that miR-10b reduced cell migration, invasion and proliferation. While inhibiting miR-155 did not correct the abnormal growth behaviour of NF1 MPNST cells, inhibiting miR-335 or enhancing let-7a expression partially corrected some abnormal growth properties of these tumour cells. These findings suggest that changes in miR-155 may represent a consequence of NF1 MPNST tumour formation, while changes in miR-335 and let-7 expression may contribute to progression of NF1 MPNSTs. These speculations are supported by the observation that *RAS* and *tenascin C* are deregulated in NF1 MPNSTs. Subsequently, these authors went on to demonstrate that miR-10b targets the *NF1* mRNA 3^′^UTR, thereby repressing the expression of neurofibromin protein. Therefore, it is suggested that miR-10b could specifically target neurofibromin to control NF1 tumorigenesis and progression. Irrespective of whether or neurofibromin is functional, as long as there is expression of the *NF1* mRNA, it is possible for the miR-10b to target its 3^′^UTR to suppress expression of neurofibromin protein. Chai et al. [38] also suggested that miR-10b could target other genes, and co-operate with other miRNAs such as miR-335 and let-7, to promote NF1 tumorigenesis and progression.

### miR-29c

Presneau et al. [39] aimed to identify a molecular target to help discriminate between neurofibromas and MPNSTs. In their study of ten matched pairs of samples of MPNSTs and neurofibromas from NF1 patients, Presneau et al. [39] showed miR-210 to have been upregulated, and a series of miRNAs to have been down-regulated (miR-30e, miR-30c, miR-340, miR-139-5p, miR-29c and Let-7g) in MPNSTs. Of these miR-29c was the most down-regulated; the gene targets of miR-29c include *COL1A1*, *COL21A1*, *COL5A2* and *TDG*, all of which were down-regulated in MPNSTs. This was confirmed using synthetic oligonucleotide mimics. Further assays showed increased migration and invasion of MPNST cells following treatment with a miR-29c mimic. Presneau et al. [39] postulated that the decreased expression levels of miR-29c are mediated by the activation of a hepatocyte growth factor receptor, *cMET*, which has been shown to be activated in MPNSTs. This hypothesis is supported by the findings of Kwiecinski et al. [57] who demonstrated that hepatocyte growth factor receptor can inhibit collagen synthesis by activating miR-29a and miR-29b. Together, these data suggest that miR-29c is regulated by the action of *cMET* which, when activated, can stimulate miR-29 to control cell migration and invasion. Thus, miR-29c can act as a tumour suppressor and could potentially be used as a means to distinguish malignant from benign tumours. This suggestion has been borne out by findings in colorectal cancer, where analysis of mRNA expression profiles has indicated that miR-29c can predict the recurrence of colorectal cancer [58], and in hepatitis B virus-related hepatocellular carcinoma, where miR29c inhibits cell proliferation and promotes apoptosis [59].

### miR-204

Recently, Gong et al. [40] reported that miR-204 contributes to the growth of MPNSTs, particularly in NF1. They postulated that this miRNA plays an important role in the carcinogenic process as it is found in cancer-associated regions of the genome and has previously been shown to be subject to high-frequency loss of heterozygosity in tumours [60]. miR-204 has also been found to act as a tumour suppressor gene in various other tumours including breast, kidney and prostate cancers [61]. This study demonstrated that the expression of miR-204 is down-regulated in tissue, which is derived from both NF1- and non-NF1-related tumours. By inducing the expression of miR-204 in MPNSTs of both NF1 and non-NF1 origin, the abnormal cellular characteristics were reversed and cell proliferation was reduced. Specifically in an NF1-related tumour cell line, induced expression of miR-204 resulted in decreased progression of malignancy and tumour development. Further bioinformatic analysis revealed that miR-204 targets *HMGA2*, a gene that regulates RAS signalling, suggesting that miR-204 may also contribute indirectly to the development of malignancy. It was also found that *HMGA2* expression is inhibited by miR-204, revealing an alternative pathway for tumour progression. It is still not clear whether miR-204 alone is sufficient to promote carcinogenesis in MPNSTs, as earlier studies have indicated that p53 inactivation and subsequent loss of expression of miR-34a may contribute to MPNST development [37]. Although these results have only been confirmed *in vitro*, miR-204 could potentially act as a biomarker for cancer diagnosis as well as an effective target for the development of therapeutic treatment.

### miR-21

In a recent study, Itani et al. [41] analysed a panel of 12 MPNSTs, 11 neurofibromas, five normal nerves and three MPNST cell lines by mRNA expression profiling. The expression of miR-21 was significantly higher in MPNSTs than in the neurofibromas or MPNST cell lines. Transfection of miR-21 inhibitor significantly increased caspase activity (*p* < 0.001) and suppressed cell growth (*p* < 0.05) while upregulating the level of PDCD4 protein. Hence, these results indicate that miR-21 is likely to play an important role in MPNST progression through its target *PDCD4*.

### NF1 microdeletions

It has been estimated that 5% of NF1 patients possess a germline 1.4-Mb microdeletion [62]. This results from the unequal recombination between two homologous NF1 low copy-number repeats [63]. NF1-microdeleted patients have an increased risk of developing MPNSTs [64]. They also appear to exhibit a more severe clinical phenotype in comparison to individuals with intragenic *NF1* mutations [65]. Analysis of the NF1 microdeleted region reveals that it contains a minimum of 16 protein coding genes, four pseudogenes and two microRNAs [66]. A recent study by Pasmant et al. [67] looked at the two miRNAs located within the 1.4-Mb microdeleted region in a set of MPNSTs and benign neurofibromas of NF1 microdeleted patients. They found that the two miRNA genes (*MIR3652* and *MIR193A* at 17q11.2 encoding miR-365-2 and miR-193a, respectively) displayed no differences in the expression between the tumour types, despite miR-193a having been previously categorized as a tumour suppressor in oral squamous cell carcinoma [68] and despite miR-365-2 having been identified as having an anti-proliferative role in colon cancer [69]. Further study will be required to assess the possible role of these two miRNAs in NF1 tumorigenesis.

### NF1-phaeochromocytomas

Tömböl et al. [70] analysed the expression pattern of miRNAs in patients with NF1-associated pheochromocytomas by real-time quantitative reverse-transcription PCR. The results of their study revealed 16 differentially expressed miRNAs; their pathway analysis suggested that Notch- and G-protein-coupled receptor signalling may be involved in tumour recurrence. These authors also demonstrated the successful use of formalin-fixed paraffin-embedded samples for the analysis of miRNAs in phaeochromocytomas.

### Epigenetic regulation of microRNAs in cancer

DNA methylation in the 5^′^ regulatory regions of genes is a major epigenetic mechanism. The role of aberrant DNA hypermethylation in the regulation of miRNA expression in human cancer has also been explored [71]. This study identified 122 miRNAs which were reported to be epigenetically regulated in 23 cancer types. Human oncomirs (miRNAs) with a role in cancer are designated as oncogenic miRNAs or oncomirs. High methylation levels compared to the protein-coding genes and at least half of the epigenetically regulated miRNAs were involved with different cancer types.

Both DNA methylation and mRNA regulation can suppress gene expression and their corresponding gene product. Su et al. [72] demonstrated that miRNAs tend to target genes with a low level of DNA methylation level in their promoter regions. They also found that miRNA target sites were significantly enriched in genes located in differentially methylated regions or partially methylated domains and that cancer genes tend to be characterized by a low level of methylation and more miRNA target sites.

### miRNAs as prognostic and diagnostic biomarkers

Dysregulation of miRNAs is fundamental to the pathogenesis of many cancers. miRNAs that regulate the expression of tumour suppressor genes and oncogenes are candidates for use in targeted therapies [73]. An improved understanding of the underlying molecular mechanisms is still required for many cancers so as to make possible the development of effective targeted therapies. Krutzfeldt et al. [74] created chemically modified antagomirs, molecules which are complementary to miRNAs and which provide a useful way of silencing specific miRNAs *in vivo*. Just as one miRNA may regulate multiple targets, one target may also be regulated by a number of different miRNAs. Hence, specificity is an important factor to consider in designing a therapeutic approach.

miRNAs are increasingly being identified as useful diagnostic and prognostic markers. One example of this is in hepatocellular carcinoma (HCC) in which it has been shown that low levels of miR-26 can be indicative of a poor prognosis [75]. These patients have also been shown to benefit from a specific type of interferon-α therapy, showing that miR-26 can be used to identify HCC patients who would be more likely to respond well to this type of treatment. miRNAs can also be shown to overcome drug resistance when targeted, in chemo-resistance [76] and resistance to anti-oestrogenic therapies [77,78], demonstrating the range of different treatment methods to which miRNAs can contribute. miRNA delivery may be used to counteract carcinogenesis. miRNAs are reliable biomarkers for testing the efficiency of chemoprevention and may be used to counteract carcinogenesis [79].

As well as individual miRNAs being able to act as biomarkers and therapeutic targets, Han et al. [80], have shown that the three genes involved in miRNA biogenesis, namely *DICER1*, *DROSHA* and *XPO5* (exportin 5), have also been shown to have therapeutic potential, at least in the case of bladder urothelial carcinoma. These genes are vital for miRNA development, and their silencing results in the inhibition of cell proliferation and apoptosis. Han et al. [80] showed that all three genes were up-regulated and had higher expression in high-grade carcinomas than low-grade carcinomas. These differential expression patterns indicate that *DICER1*, *DROSHA* and *XPO5* could play active roles in carcinogenesis.

The use of miRNAs as useful biomarkers of tumorigenesis is supported by the finding that they are secreted into bodily fluids [81] and, hence, are circulating around the body so that they can be easily and accurately observed by means of microarray and quantitative PCR methods, allowing their detection to be both sensitive and specific [82]. There are already clinical diagnostic tests available that use miRNAs as biomarkers, such as the ProOnc TumorSource Dx, a proprietary test offered by Prometheus Labs (San Diego, CA, USA) (http://www.prometheuslabs.com), that determines the expression levels of 48 different miRNA biomarkers in a tissue sample, from which the results are used to determine the tissue of origin of metastatic cancer.

## Conclusions

The discovery of miRNAs nearly 20 years ago introduced us to a new regulatory mechanism enabling a better understanding of the molecular pathogenesis of cancers. Dysregulation of miRNAs is fundamental to the pathogenesis of many cancers. miRNAs have the potential to target as many as several hundred genes simultaneously making them attractive prognostic biomarkers and therapeutic targets in cancer. miRNAs are not only involved in tumour progression, but also play a role in cancer invasion, metastasis, epigenetic alterations, chemo-resistance and radio-resistance. Although research on the role of miRNAs in NF1 tumorigenesis is still in its infancy, the experimental data obtained so far indicate that a number of miRNAs may be involved in NF1 tumorigenesis, including miR-29c, miR-34a, miR-214, miR-10b, miR-204 and miR-21. However, we lack an understanding of how different miRNAs identified in NF1 tumours might interact with neurofibromin and other tumour-associated proteins and whether these miRNAs can be linked to specific cancer signalling pathways. miRNAs involved in regulating the expression of oncogenes and tumour suppressor genes are candidates for targeted therapy for NF1 tumours. Therefore, it is hoped that the study of these small RNAs will eventually make a significant difference in treating NF1, at least to delay, if not entirely eliminate, the onset of tumorigenesis. The identification not only of differentially expressed microRNAs in tumours but also their target genes is providing new avenues for therapeutic approaches. As with any new therapeutic approach, there are of course many obstacles to be overcome before miRNAs can be used in clinical practise. However, it appears to present a very promising therapeutic avenue.

## Competing interests

The authors declare that they have no competing interests.

## Authors’ contributions

AS contributed to the compilation of the first draft, DNC and MU improved the manuscript. All authors read and approved the final manuscript.

## References

[B1] Ambros V The functions of animal microRNAs Nature 2001 431 350 355 15372042 10.1038/nature02871

[B2] Bartel DP MicroRNAs: genomics, biogenesis, mechanism and function Cell 2004 116 281 297 10.1016/S0092-8674(04)00045-5 14744438

[B3] Lai EC MicroRNAs are complementary to 3′UTR sequence motifs that mediate negative post-transcriptional regulation Nat Genet 2002 30 363 364 10.1038/ng865 11896390

[B4] Lee RC Feinbaum RL Ambros V The C. elegans heterochronic gene lin-4 encodes small RNAs with antisense complementarity to lin-14 Cell 1993 75 843 854 10.1016/0092-8674(93)90529-Y 8252621

[B5] Reinhart BJ Slack FJ Basson M Pasquinelli AE Bettinger JC Rougvie AE Horvitz HR Ruvkun G The 21-nucleotide let-7 RNA regulates developmental timing in Caenorhabtidis elegans Nature 2000 403 901 906 10.1038/35002607 10706289

[B6] Pasquinelli AE Reinhart BJ Slack FJ Martindale MQ Kuroda MI Maller B Hayward DC Ball EE Degnan B Muller P Spring J Srinivasan A Fishman M Finnerty J Corbo J Levine M Leahy P Davidson E Ruvkun G Conservation of the sequence and temporal expression of Let-7 heterochronic regulatory RNA Nature 2000 408 86 89 10.1038/35040556 11081512

[B7] Yu J Wang F Yang GH Wang FL Ma YN Du ZW Zhang JW Human microRNA clusters: genomic organization and expression profile in leukemia cell lines Biochem Biophys Res Commun 2006 13 59 68 16934749 10.1016/j.bbrc.2006.07.207

[B8] Sayed D Abdellatif M MicroRNAs in development and disease Physiol Rev 2011 91 827 887 10.1152/physrev.00006.2010 21742789

[B9] Ladomery MR Maddocks DG Wilson ID MicroRNAs: their discovery, biogenesis, function and potential use as biomarkers in non-invasive prenatal diagnostics Int J Mol Epidemiol Genet 2011 2 253 260 21915364 PMC3166153

[B10] Lee Y Jeon K Lee JT Kim S Kim VN MicroRNA maturation: stepwise processing and subcellular localisation EMBO J 2002 23 4051 4060 12198168 10.1093/emboj/cdf476 PMC126204

[B11] Zeng Y Yi R Cullen BR Recognition and cleavage of primary microRNA precursors by the nuclear processing enzyme Drosha EMBO J 2005 24 138 148 10.1038/sj.emboj.7600491 15565168 PMC544904

[B12] Denli AM Tops BBJ Plasterk RHA Ketting RF Hannon GJ Processing of primary microRNAs by the microprocessor complex Nature 2004 432 231 235 10.1038/nature03049 15531879

[B13] Gregory RI Yan KP Amuthan G Chendrimada T Doratotaj B Cooch N Shiekhatter R The microprocessor complex mediates the genesis of microRNAs Nature 2004 432 235 240 10.1038/nature03120 15531877

[B14] Landthaler M Yalcin A Tuschl T The human DiGeorge syndrome critical region gene 8 and its D. melanogaster homolog are required for miRNA biogenesis Curr Biol 2004 14 2162 2167 10.1016/j.cub.2004.11.001 15589161

[B15] Yi R Qin Y Macara IG Cullen BR Exportin-5 mediates the nuclear export of pre-microRNAs and short hairpin RNAs Genes Dev 2003 17 3011 3016 10.1101/gad.1158803 14681208 PMC305252

[B16] Lund E Guttinger S Calado A Dahlberg JE Kutay U Nuclear export of microRNA precursors Science 2004 303 95 98 10.1126/science.1090599 14631048

[B17] Hwang HW Mendell JT MicroRNAs in cell proliferation, cell death and tumourigenesis Br J Cancer 2006 94 776 780 10.1038/sj.bjc.6603023 16495913 PMC2361377

[B18] Matranga C Tomari Y Shin C Bartel DP Zamore PD Passenger-strand cleavage facilitates assembly of siRNA into Ago2-containing RNAi enzyme complexes Cell 2005 123 607 620 10.1016/j.cell.2005.08.044 16271386

[B19] Winter J Jung S Keller S Gregory RI Diederichs S Many roads to maturity: microRNA biogenesis pathways and their regulation Nat Cell Biol 2009 11 228 234 10.1038/ncb0309-228 19255566

[B20] Tan G Shi Y Wu ZH MicroRNA-22 promotes cell survival upon UV radiation by repressing PTEN Biochem Biophys Res Commun 2012 2012 417 546 551 22166214 10.1016/j.bbrc.2011.11.160 PMC3259290

[B21] Hawkins PG Morris KV RNA and transcriptional modification of gene expression Cell Cycle 2008 7 602 607 10.4161/cc.7.5.5522 18256543 PMC2877389

[B22] Esquela-Kerscher A Slack FJ Oncomirs-microRNAs with a role in cancer Nat Rev Cancer 2006 6 259 269 10.1038/nrc1840 16557279

[B23] Hammond SM MicroRNA detection comes of age Nat Methods 2006 3 12 13 10.1038/nmeth0106-12 16369545

[B24] Chen C Ridzon DA Broomer AJ Zhou Z Lee DH Nguyen JT Barbisin M Xu NL Mahuvakar VR Andersen MR Lao KQ Livak KJ Guegler KJ Real-time quantification of microRNAs by stem-loop RT-PCR Nucleic Acids Res 2005 33 e179 10.1093/nar/gni178 16314309 PMC1292995

[B25] Liu CG Calin GA Meloon B Gamliel N Sevignani C Ferracin M Dumitru CD Shimizu M Zupo S Dono M Alder H Bullrich F Negrini M Croce CM An oligonucleotide microchip for genome-wide microRNA profiling in human and mouse tissues Proc Nat Acad Sci USA 2004 101 9740 9744 10.1073/pnas.0403293101 15210942 PMC470744

[B26] Neely LA Patel S Garver J Gallo M Hackett M McLaughlin S Nadel M Harris J Gullans S Rooke J A single-molecule method for the quantitation of microRNA gene expression Nat Methods 2006 3 41 46 10.1038/nmeth825 16369552

[B27] Lu J Getz G Miska EA Alvarez-Saavedra E Lamb J Peck D Sweet-Cordero A Ebert BL Mak RH Ferrando AA Downing JR Jacks T Horvitz HR Golub TR MicroRNA expression profiles classify human cancers Nature 2005 435 834 838 10.1038/nature03702 15944708

[B28] Rosenfeld N Aharonov R Meiri E Rosenwald S Spector Y Zepeniuk M Benjamin H Shabes N Tabak S Levy A Lebanony D Goren Y Silberschein E Targan N Ben-Ari A Gilad S Sion-Vardy N Tobar A Feinmesser M Kharenko O Nativ O Nass D Perelman M Yosepovich A Shalmon B Polak-Charcon S Fridman E Avniel A Bentwich I MicroRNAs accurately identify cancer tissue origin Nat Biotechnol 2008 26 462 469 10.1038/nbt1392 18362881

[B29] Slaby O Bienertova-Vasku J Svoboda M Vyzula R Genetic polymorphisms and microRNAs: new direction in molecular epidemiology of solid cancer J Cell Mol Med 2012 16 8 21 10.1111/j.1582-4934.2011.01359.x 21692980 PMC3823089

[B30] Gong J Tong Y Zhang HM Wang K Hu T Shan G Sun J Guo AY Genome-wide identification of SNPs in microRNA genes and the SNP effects on microRNA target binding and biogenesis Hum Mutat 2012 33 254 263 10.1002/humu.21641 22045659

[B31] Zhang L Huang J Yang N Greshock J Megraw MS Giannakakis A Liang S Naylor TL Barchetti A Ward MR Yao G Medina A O'brien-Jenkins A Katsaros D Hatzigeorgiou A Gimotty PA Weber BL Coukos G microRNAs exhibit high frequency genomic alterations in human cancer Proc Natl Acad Sci USA 2006 103 9136 9141 10.1073/pnas.0508889103 16754881 PMC1474008

[B32] Marcinkowska M Szymanski M Krzyzosiak WJ Kozlowski P Copy number variation of microRNA genes in the human genome BMC Genomics 2011 12 183 10.1186/1471-2164-12-183 21486463 PMC3087710

[B33] Upadhyaya M Genetic basis of tumorigenesis in NF1 malignant peripheral nerve sheath tumors Front Biosci 2011 16 937 951 10.2741/3727 21196210

[B34] Cheng AM Byrom MW Shelton J Ford LP Antisense inhibition of human miRNAs and indications for involvement of miRNA in cell growth and apoptosis Nucleic Acids Res 2005 33 1290 1297 10.1093/nar/gki200 15741182 PMC552951

[B35] Cimmino A Calin GA Fabri M Iorio MV Ferracin M Shimizu M Wojcik SE Aqelin RI Zupo S Dono M Rassenti L Alder H Volinia S Liu CG Kipps TJ Negrini M Croce CM miR-15 and miR-16 induce apoptosis by targeting BCL2 Proc Natl Acad Sci USA 2005 102 13944 13949 10.1073/pnas.0506654102 16166262 PMC1236577

[B36] Calin GA Sevignani C Dumitru CD Hyslop T Noch E Yendamuri S Shimizu M Rattan S Bullrich F Negrini M Croce CM Human microRNA genes are frequently located at fragile sites and genomic regions involved in cancers Proc Natl Acad Sci USA 2004 101 2999 3004 10.1073/pnas.0307323101 14973191 PMC365734

[B37] Subramanian S Thayanithy V West RB Lee CH Beck AH Zhu S Downs-Kelly E Montgomery K Goldblum JR Hogendoorn PC Corless CL Oliveira AM Dry SM Nielsen TO Rubin BP Fletcher JA Fletcher CD van de Rijn M Genome-wide transcriptome analyses reveal p53 inactivation mediated loss of miR-34a expression in malignant peripheral nerve sheath tumours J Pathol 2010 220 58 70 10.1002/path.2633 19890883 PMC4058327

[B38] Chai G Liu N Ma J Li H Oblinger JL Prahalad AK Gong M Chang LS Wallace M Muir D Guha A Phipps RJ Hock JM Yu X MicroRNA-10b regulates tumourigenesis in neurofibromatosis type 1 Cancer Sci 2010 101 1997 2004 10.1111/j.1349-7006.2010.01616.x 20550523 PMC11159772

[B39] Presneau N Eskandarpour MS Henderson S Halai D Tirabosco R Flanagan AM Micro-RNA profiling of peripheral nerve sheath tumours identifies miR-29c as a tumour suppressor gene involved in tumour progression Br J Cancer 2012 10.1038/bjc.2012.518 PMC3590650 23175151

[B40] Gong M Ma J Li M Zhou M Hock JM Yu X MicroRNA-204 critically regulates carcinogenesis in malignant peripheral nerve sheath tumors Neuro Oncol 2012 14 1007 1017 10.1093/neuonc/nos124 22718995 PMC3408257

[B41] Itani S Kunisada T Morimoto Y Yoshida A Sasaki T Ito S Ouchida M Sugihara S Shimizu K Ozaki T MicroRNA-21 correlates with tumorigenesis in malignant peripheral nerve sheath tumor (MPNST) via programmed cell death protein 4 (PDCD4) J Cancer Res Clin Oncol 2012 138 1501 1509 10.1007/s00432-012-1223-1 22526161 PMC11824360

[B42] Lee Ming J Choji H Galas DJ Wang K The systems biology of neurofibromatosis type 1 - critical roles for microRNA Exp Neurol 2012 235 464 464 10.1016/j.expneurol.2011.10.023 22075182

[B43] Lammens T Li J Leone G De Veylder L Atypical E2Fs: new players in the E2F transcription factor family Trends Cell Biol 2009 19 111 118 10.1016/j.tcb.2009.01.002 19201609 PMC2808192

[B44] Reimer D Sadr S Wiedemair A Stadlmann S Concin N Hofstetter G Müller-Holzner E Marth C Zeimet AG Clinical relevance of E2F family members in ovarian cancer - an evaluation in a training set of 77 patients Clin Cancer Res 2007 13 144 151 10.1158/1078-0432.CCR-06-0780 17200349

[B45] Yin D Ogawa S Kawamata N Leiter A Ham M Li D Doan NB Said JW Black KL Koeffler PH miR-34a functions as a tumor suppressor modulating EGFR in glioblastoma multiforme Oncogene 2012 10 1038 10.1038/onc.2012.132 PMC4085050 22580610

[B46] Li L Yuan L Luo J Gao J Guo J Xie X MiR-34a inhibits proliferation and migration of breast cancer hrough down-regulation of Bcl-2 and SIRT1 Clin Exp Med 2012 10.1007/s10238-012-0186-5 22623155

[B47] Kumar B Yadav A Lang J Teknos TN Kumar P Dysregulation of microRNA-34a expression in head and neck squamous cell carcinoma promotes tumor growth and tumor angiogenesis PLoS One 2012 7 e37601 10.1371/journal.pone.0037601 22629428 PMC3358265

[B48] Yan K Gao J Yang T Ma Q Qiu X Fan Q Ma B MicroRNA-34a inhibits the proliferation and metastasis of osteosarcoma cells both in vitro and in vivo PLoS One 2012 7 e33778 10.1371/journal.pone.0033778 22457788 PMC3310405

[B49] Schwarzenbach H Milde-Langosch K Steinbach B Müller V Pantel K Diagnostic potential of PTEN-targeting miR-214 in the blood of breast cancer patients Breast Cancer Res Treat 2012 134 933 941 10.1007/s10549-012-1988-6 22350790

[B50] Yang H Kong W He L Zhao JJ O'Donnell JD Wang J Wenham RM Coppola D Kruk PA Nicosia SV Cheng JQ MicroRNA expression profiling in human ovarian cancer: miR-214 induces cell survival and cisplatin resistance by targeting PTEN Cancer Res 2008 68 425 433 10.1158/0008-5472.CAN-07-2488 18199536

[B51] Peng RQ Wan HY Li HF Liu M Li X Tang H MicroRNA-214 suppresses growth and invasiveness of cervical cancer cells by targeting UDP-N-acetyl-α-d-galactosamine:polypeptide N-acetylgalactosaminyltransferase 7 J Biol Chem 2012 287 14301 14309 10.1074/jbc.M111.337642 22399294 PMC3340176

[B52] Shih TC Tien YJ Wen CJ Yeh TS Yu MC Huang CH Lee YS Yen TC Hsieh SY MicroRNA-214 downregulation contributes to tumor angiogenesis via inducing secretion of hepatoma-derived growth factor in human hepatoma J Hepatol 2012 57 584 591 10.1016/j.jhep.2012.04.031 22613005

[B53] Lee YB Bantounas I Lee DY Phylactou L Caldwell MA Uney JB Twist-1 regulates the miR-199a/214 cluster during development Nucleic Acids Res 2009 1 123 128 19029138 10.1093/nar/gkn920 PMC2615617

[B54] Tanzer A Amemiya CT Kim CB Stadler PF Evolution of microRNAs located within Hox gene clusters J Exp Zool B Mol Dev Evol 2005 304 75 85 15643628 10.1002/jez.b.21021

[B55] Garzon R Garofalo M Martelli MP Briesewitz R Wang L Fernandez-Cymering C Volinia S Liu CG Schnittger S Haferlach T Liso A Diverio D Mancini M Meloni G Foa R Martelli MF Mecucci C Croce CM Falini B Distinctive microRNA signature of acute myeloid leukemia bearing cytoplasmic mutated nucleophosmin Proc Natl Acad Sci USA 2008 105 3945 3950 10.1073/pnas.0800135105 18308931 PMC2268779

[B56] Ma L Teruya-Feldstein J Weinberg RA Tumour invasion and metastasis initiated by microRNA-10b in breast cancer Nature 2007 449 682 688 10.1038/nature06174 17898713

[B57] Kwiecinski M Noetel A Elfimova N Trebicka J Schievenbusch S Strack I Molnar L von Brandenstein M Töx U Nischt R Coutelle O Dienes HP Odenthal M Hepatocyte growth factor (HGF) inhibits collagen I and IV synthesis in hepatic stellate cells by miRNA-29 induction PLoS One 2011 6 e24568 10.1371/journal.pone.0024568 21931759 PMC3170366

[B58] Kuo TY Hsi E Yang IP Tsai PC Wang JY Juo SH Computational analysis of mRNA expression profiles identifies microRNA-29a/c as predictor of colorectal cancer early recurrence PLoS One 2012 7 e31587 10.1371/journal.pone.0031587 22348113 PMC3278467

[B59] Wang CM Wang Y Fan CG Xu FF Sun WS Liu YG Jia JH miR-29c targets TNFAIP3, inhibits cell proliferation and induces apoptosis in hepatitis B virus-related hepatocellular carcinoma Biochem Biophys Res Commun 2011 411 586 592 10.1016/j.bbrc.2011.06.191 21763284

[B60] Bauer VL Braselmann H Henke M Mattern D Walch A Unger K Baudis M Lassmann S Huber R Wienberg J Werner M Zitzelsberger HF Chromosomal changes characterize head and neck cancer with poor prognosis J Mol Med 2008 86 1353 1365 10.1007/s00109-008-0397-0 18810378

[B61] Wang FE Zhang C Maminishkis A Dong L Zhi C Li R Zhao J Majerciak V Gaur AB Chen S Miller SS MicroRNA-204/211 alters epithelial physiology FASEB J 2010 24 1552 1571 10.1096/fj.08-125856 20056717 PMC3231816

[B62] Kluwe L Siebert R Gesk S Friedrich RE Tinschert S Kehrer-Sawatzki H Mautner VF Screening 500 unselected neurofibromatosis 1 patients for deletions of the NF1 gene Hum Mutat 2004 23 111 116 10.1002/humu.10299 14722914

[B63] Dorschner MO Sybert VP Weaver M Pletcher BA Stephens K NF1 microdeletion breakpoints are clustered at flanking repetitive sequences Hum Mol Genet 2000 9 35 46 10.1093/hmg/9.1.35 10587576

[B64] De Raedt T Brems H Lopez-Correa C Vermeesch JR Marynen P Legius E Genomic organization and evolution of the NF1 microdeletion region Genomics 2004 84 346 360 10.1016/j.ygeno.2004.03.006 15233998

[B65] Upadhyaya M Ruggieri M Maynard J Osborn M Hartog C Mudd S Penttinen M Cordeiro I Ponder M Ponder BAJ Krawczak M Cooper DN Gross deletions of the neurofibromatosis type 1 (NF1) gene are predominantly of maternal origin and commonly associated with a learning disability, dysmorphic features and developmental delay Hum Genet 1998 102 591 597 10.1007/s004390050746 9654211

[B66] Jenne DE Tinschert S Dorschner MO Hameister H Stephens K Kehrer-Sawatzki H Complete physical map and gene content of the human NF1 tumour suppressor region in human and mouse Genes Chrom Cancer 2003 37 111 120 10.1002/gcc.10206 12696059

[B67] Pasmant E Masliah-Planchon J Lévy P Laurendeau I Ortonne N Parfait B Valeyrie-Allanore L Leroy K Wolkenstein P Vidaud M Vidaud D Bièche I Identification of genes potentially involved in the increased risk of malignancy in NF1-microdeleted patients Mol Med 2011 17 79 87 20844836 10.2119/molmed.2010.00079 PMC3022985

[B68] Kozaki K Imoto I Mogi S Omura K Inazawa J Exploration of tumour-suppressive microRNAs silenced by DNA hypermethylation in oral cancer Cancer Res 2008 68 2094 2105 10.1158/0008-5472.CAN-07-5194 18381414

[B69] Nie J Liu L Zheng W Chen L Wu X Xu Y Du X Han W microRNA-365, down-regulated in colon cancer, inhibits cell cycle progression and promotes apoptosis of colon cancer cells by probably targeting cyclin D1 and Bcl-2 Carcinogenesis 2012 33 220 225 10.1093/carcin/bgr245 22072615

[B70] Tömböl Z Eder K Kovács A Szabó PM Kulka J Likó I Zalatnai A Rácz G Tóth M Patócs A Falus A Rácz K Igaz P MicroRNA expression profiling in benign (sporadic and hereditary) and recurring adrenal pheochromocytomas Mod Pathol 2010 23 1583 1595 10.1038/modpathol.2010.164 20818339

[B71] Kunej T Godnic I Ferdin J Horvat S Dovc P Calin GA Epigenetic regulation of microRNAs in cancer: an integrated review of literature Mutat Res 2011 717 77 84 10.1016/j.mrfmmm.2011.03.008 21420983

[B72] Su Z Xia J Zhao Z Functional complementation between transcriptional methylation regulation and post-transcriptional microRNA regulation in the human genome BMC Genomics 2011 5 S15 22369656 10.1186/1471-2164-12-S5-S15 PMC3287497

[B73] Nana-Sinkam SP Croce CM MicroRNAs as therapeutic targets in cancer Transl Res 2011 157 216 225 10.1016/j.trsl.2011.01.013 21420032

[B74] Krutzfeldt J Rajewsky N Braich R Rajeev KG Tuschl T Manoharan M Stoffel M Silencing of microRNAs in vivo with ‘antagomirs’ Nature 2005 438 685 689 10.1038/nature04303 16258535

[B75] Ji J Shi J Budhu A Yu Z Forgues M Roessler S Ambs S Chen Y Meltzer PS Croce CM Qin LX Man K Lo CM Lee J Ng IO Fan J Tang ZY Sun HC Wang XW MicroRNA expression, survival, and response to interferon in liver cancer N Engl J Med 2009 361 1437 1447 10.1056/NEJMoa0901282 19812400 PMC2786938

[B76] Meng F Henson R Lang M Wehbe H Maheshwari S Mendell JT Jiang J Schmittgen TD Patel T Involvement of human micro-RNA in growth and response to chemotherapy in human cholangiocarcinoma cell lines Gastroenterology 2006 2006 130 2113 2129 16762633 10.1053/j.gastro.2006.02.057

[B77] Miller TE Ghoshal K Ramaswamy B Roy S Datta J Shapiro CL Jacob S Majumder S MicroRNA-221/222 confers tamoxifen resistance in breast cancer by targeting p27Kip1 J Biol Chem 2008 283 29897 29903 10.1074/jbc.M804612200 18708351 PMC2573063

[B78] Zhao JJ Lin J Yang H Kong W He L Ma X Coppola D Cheng JQ MicroRNA-221/222 negatively regulates estrogen receptor alpha and is associated with tamoxifen resistance in breast cancer J Biol Chem 2008 283 31079 31086 10.1074/jbc.M806041200 18790736 PMC2576549

[B79] Izzotti A Molecular medicine and the development of cancer chemopreventive agents Ann N Y Acad Sci 2012 1259 26 32 10.1111/j.1749-6632.2012.06646.x 22758633

[B80] Han Y Liu Y Gui Y Cai Z Inducing cell proliferation inhibition and apoptosis via silencing Dicer, Drosha, and Exportin 5 in urothelial carcinoma of the bladder J Surg Oncol 2012 10.1002/jso.23214 22766726

[B81] Steer CJ Subramanian S Circulating microRNAs as biomarkers: a new frontier in diagnostics Liver Transpl 2012 18 265 269 10.1002/lt.23377 22228587

[B82] Ajit SK Circulating microRNAs as biomarkers, therapeutic targets, and signaling molecules Sensors (Basel) 2012 12 3359 3369 10.3390/s120303359 22737013 PMC3376561

